# Intra-articular vs. systemic administration of etanercept in antigen-induced arthritis in the temporomandibular point. Part I: histological effects

**DOI:** 10.1186/1546-0096-7-5

**Published:** 2009-02-06

**Authors:** Kasper D Kristensen, Peter Stoustrup, Annelise Küseler, Thomas K Pedersen, Jens R Nyengaard, Ellen Hauge, Troels Herlin

**Affiliations:** 1 Department of Orthodontics, Faculty of Health Sciences, University of Aarhus, Vennelyst Boulevard 9, 8000 Aarhus C, Denmark; 2 Stereology and Electron Microscopy Research Laboratory and MIND Center, University of Aarhus, Ole Worms Allé, bygning 1184, 8000 Aarhus C, Denmark; 3 Department of Rheumatology, Aarhus University Hospital, Nørrebrogade 44, 8000 Aarhus C, Denmark; 4 Department of Pediatrics, Aarhus University Hospital, Brendstrupgaardvej 100, 8200 Aarhus N, Denmark

## Abstract

**Background:**

Temporomandibular joint (TMJ) arthritis in children causes alterations in craniomandibular growth. This abnormal growth may be prevented by an early anti-inflammatory intervention. We have previously shown that intra-articular (IA) corticosteroid reduces TMJ inflammation, but causes concurrent mandibular growth inhibition in young rabbits. Blockage of TNF-α has already proven its efficacy in children with juvenile idiopathic arthritis not responding to standard therapy. In this paper we evaluate the effect of IA etanercept compared to subcutaneous etanercept in antigen-induced TMJ-arthritis in rabbits on histological changes using histomorphometry and stereology. This article presents the data and discussion on the anti-inflammatory effects of systemic and IA etanercept. In Part II the data on the effects of systemic and IA etanercept on facial growth are presented.

**Methods:**

Forty-two rabbits (10 weeks old) pre-sensitized with ovalbumin and locally induced inflammation in the temporomandibular joints were divided into three groups: a placebo group receiving IA saline injections in both joints one week after arthritis induction (n = 14), an IA etanercept group receiving 0.1 mg/kg etanercept per joint one week after arthritis induction (n = 14) and a systemic etanercept group receiving 0.8 mg/kg etanercept weekly throughout the 12-week study (n = 14). Arthritis was maintained by giving four inductions three weeks apart. Additional IA saline or etanercept injections were also given one week after the re-inductions. Histomorphometric and unbiased stereological methods (optical fractionator) were used to assess and estimate the inflammation in the joints.

**Results:**

The histomorphometry showed synovial proliferation in all groups. The plasma cell count obtained by the optical fractionator was significantly reduced when treating with systemic etanercept but not with IA etanercept. Semi-quantitative assessments of synovial proliferation and subsynovial inflammation also showed reduced inflammation in the systemic etanercept group. However, the thickness of the synovial lining and volume of the subsynovial connective tissue showed no differences between the groups.

**Conclusion:**

An anti-inflammatory effect of systemic etanercept on the synovial tissues in the temporomandibular joint was shown. However, IA etanercept at the given dose had no significant effect on the severity of chronic inflammation on the parameters here tested in ovalbumin antigen-induced arthritis.

## Background

In juvenile idiopathic arthritis (JIA) the temporomandibular joints (TMJs) are affected in more than 60% of the patients, depending on the examination method used [1-3]. TMJ involvement can lead to serious mandibular growth alterations [4-6] because of a unique intracapsular localization of the mandibular growth zone in the condylar cartilage [7]. The use of intra-articular (IA) corticosteroids in the TMJ shows a favourable response in more than 50% by improving jaw movement and reducing pain [8]. However, the clinical long-term effects on growth of IA corticosteroid therapy in juvenile TMJs are unknown. In experimental TMJ arthritis we demonstrated that IA corticosteroid reduced inflammation but accentuated mandibular growth disturbances [9-11]. Therefore a search for alternative local anti-inflammatory treatment is warranted.

The efficacy and safety of etanercept, a soluble tumor necrosis factor (TNF)-α receptor fusion protein, has been proven in a randomized controlled withdrawal-study in children with severe polyarticular JIA [12,13]. In a small clinical trial, administration of IA etanercept and IA corticosteroid shows comparable results in knee joints of rheumatoid arthritis patients [14]. The effects in this study were measured using questionnaires and ultrasonography. To our knowledge no data exist on histology in IA etanercept. The use of IA etanercept is a promising candidate of local inflammation control in the TMJs, since no side effects are expected in reducing the mandibular growth.

In this study we investigated the effect of IA and systemic administration of etanercept in ovalbumin antigen-induced arthritis in the TMJ in young growing rabbits. The effects on inflammation control will be discussed in the present article and the aspects on mandibular growth will be discussed in Part II: Mandibular growth [15].

## Materials and methods

Forty-two female 10-week-old New Zealand white rabbits (*Oryctolagus cuniculus*) were used. The rabbits were housed at the animal facilities at Aarhus University, Denmark, and had constant access to food and water. The animal-welfare was supervised daily by the staff at the housing-facilities. All procedures in this study were approved by the Danish ethical committee for experimental animal research.

**Figure 1:**
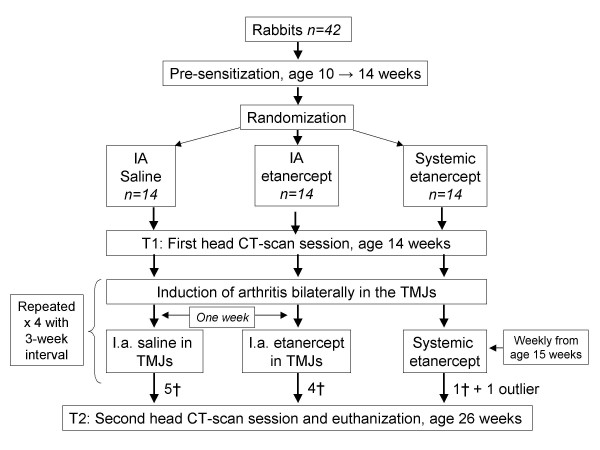
**Flow chart of the study design**. Induction of arthritis was performed four times with three weeks intervals in all three groups. Number of animals lost during the study is shown with a †.

The study design is shown in figure 1. All rabbits were sensitized to ovalbumin using the method described by Kapila [16], using 1 ml of ovalbumin (1 mg/ml) (Sigma Chemicals, Steinheim, Germany) dissolved in physiological saline and Freund's complete adjuvant^® ^(IFA; Sigma Chemicals, St. Louis, MO, USA). The procedure was repeated two weeks later using Freund's incomplete adjuvant^®^. All animals were tested for sensitivity one week after by subcutaneous injection of 1 ml (1 mg/ml) ovalbumin on a shaved spot (3 × 3 cm) on the back. Injection-site inspection was done twelve hours later for signs of local inflammation. An animal was considered sensitized if the erythema was more than 1 × 1 cm, and the induration was more than 0.5 × 0.5 cm.

Arthritis was induced in both TMJs one week after sensitation by an IA injection with sterile filtered 0.1 ml ovalbumin (10 mg/ml) dissolved in physiological saline. Arthritis was induced a total of four times, with 3-week intervals in all animals. At the fourth arthritis induction the ovalbumin dosage was reduced to 5 mg/ml because of anaphylactic reactions at the 10 mg/ml dose. All IA injections in this study were done under anesthesia with 16 mg ketamine, 0.8 mg xylazine and 0.17 mg acepromazine given subcutaneously. The IA injections were done as described by Kapila [16]. The mandible was manipulated under general anesthesia and the condylar head was easily palpated. When inside the lower joint chamber the injection is done slowly.

Animals were randomly assigned to receive either IA saline (2 × 0.1 ml), IA etanercept (Enbrel^®^, Wyeth Pharmaceuticals Inc., Collegeville, PA, USA) bilaterally (2 × 0.1 mg/kg) or systemic etanercept (0.8 mg/kg) treatment.

The etanercept dose is calculated as follows:

$$\begin{array}{l} \text{Desired concentration}(mg/kg)=\\ \frac{\text{Amount of injected fluid}(ml)\cdot \text{Injected etanercept concentration}(mg/ml)}{\text{Average weight of animals}(kg)} \end{array}$$

IA saline or IA etanercept were administered one week after every arthritis induction. We administered etanercept systemically in the last group once weekly beginning one week after the first arthritis induction. A total of ten animals died before study completion because of anaphylactic shock against the ovalbumin at the 10 mg/ml dose, which prompted dose reduction as mentioned.

Four days after the last treatment, the animals were euthanized for retrieval of histological samples using intravenous pentobarbital (200 mg/ml).

### Histological samples

One randomly chosen TMJ from each animal was retrieved en bloc and placed in 70% ethanol. Dehydration was done in 96% ethanol, 99% ethanol and equal amounts of ethanol and acetone for four days.

A vertical axis perpendicular to the condylar cartilage was defined. The joints were embedded in methylmethacrylate (MMA, Merck KgaA, Darmstadt, Germany) with the condylar cartilage parallel to the table. Before sectioning, the joints were given a systematic randomly rotation around the vertical axis. The sectioning was performed on a microtome (Reichert-Jung Polycut E, Heidelberg, Germany) parallel to the vertical axis using heavy-duty knives (Leica Microsystems GmbH, Wetzlar, Germany), and sectioning of the entire condyle was initiated when condylar cartilage was recognized and continued throughout the condyle resulting in five to eleven sampling levels. Sections were sampled in a systematic and uniformly random manner in cycles of four consecutive 25 μm sections followed by four consecutive 6.5 μm sections separated by 751 μm (25 × 4 + 6.5 × 4 + 25 × 25 μm). The 6.5 μm sections were placed on gelatine-coated slides and stained with Goldner-Trichrome. The 25 μm sections were stained floating with toluidin-blue and mounted on slides.

The microtome was calibrated to verify the exact distance between the sections. The microtome advance was 23.9 μm when the microtome was set to cut 25 μm thick sections [17]. All sections were evaluated by a blinded observer.

### Conventional histomorphometry

**Figure 2:**
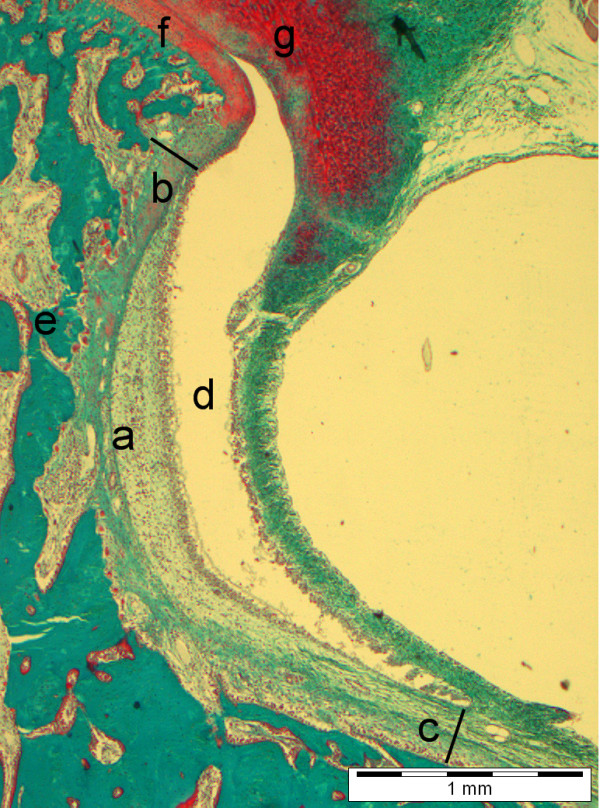
***a*: The subsynovial connective tissue (SSCT), *b*: upper limit of SSCT, *c*: lower limit of SSCT, *d*: lower joint chamber, *e*: condylar bone, *f*: condylar cartilage, *g*: discus articularis (Goldner-trichrome)**. From [9].

A central section from each joint was evaluated by conventional histomorphometry. The parameters of interest were: fractional surface, synovial lining thickness, semi-quantitative (S-Q) scoring of inflammation in the sub-synovial connective tissue (SSCT), and synovial proliferation. The SSCT is defined as the connective tissue between the synovial lining and the mandibular bone in the lower joint chamber in the TMJ. The lower border is an artificial line perpendicular from the lower synovial fold to the condylar bone and the upper border is the condylar cartilage. This area is easily recognized in the microscope (Figure 2).

The fractional surface is the percentage of normal synovial lining. The fractional surface measurements were done in an Olympus BX 51 microscope (magnification 200×) by a single observer and an Olympus standard line-grid (Olympus Denmark, Ballerup, Denmark). We counted the intersections with normal synovial lining, hyperplastic synovial lining and villous hyperplasia in the lower joint-chamber. A mean of 138 intersections were counted in each joint. Both hyperplastic synovial lining and villous hyperplasia are considered pathological features, and the statistical evaluation was therefore done on the fraction of normal synovial lining.

Synovial thickness was measured by orthogonal intersections using the computer program SigmaScan Pro (SigmaScan Pro 4.03, Image Analysis, SPSS Inc., Chicago, IL, USA). An attached digitizer and the Olympus standard line grid was randomly rotated over the synovial lining (magnification 200×). For each section 90–200 distances were measured on one section. A mean value per section (i.e. joint) was calculated, and this was multiplied by π/4 for correction of the oblique cutting phenomenon [18].

An experienced histologist scored the inflammation degree in the SSCT by plasma cell presence blindedly. The degree of inflammation was given a score from 0 to 4. Scoring criteria: 0 = no plasma cells; 1 = few, scattered; 2 = clearly present; 3 = multiple plasma cells organized in bands; 4 = massive presence of plasma cells. Synovial proliferation was scored accordingly from 0 to 4. Scoring criteria: 0 = none; 1 = small areas with synovial proliferation; 2 = large areas with synovial proliferation; 3 = invasion into the joint cavity by synovial proliferation; 4 = joint cavity totally occupied by synovial proliferation. A score was made of both the inner and outer synovial linings and the combined score was divided by two.

### Stereological method application

We used Cavalieri's unbiased volume estimator [19] on the 6.5 μm sections to estimate the volume of the SSCT. Point counting was only done in sections where synovial cells could be defined. The area was calculated using stereology software (CAST version 2, Visiopharm, Horsholm, Denmark) that projected an unbiased counting grid on the microscope image. A total magnification of 102× was used and the area per point was 7500 μm^2^.

By applying stereological methods 3D information can be obtained from correctly sampled 2D sections. This enables an accurate, unbiased quantification. The optical fractionator was used for plasma cell counting in the SSCT on the 25 μm sections [17,20]. By using the optical fractionator, an unbiased estimate of the exact number of plasma cells in the SSCT can be calculated, producing an estimate of the severity of the chronic inflammation [21]. When using the optical fractionator with varying height sampling fraction the local changes in height (shrinkage) of the sections is taken into consideration [17].

**Figure 3:**
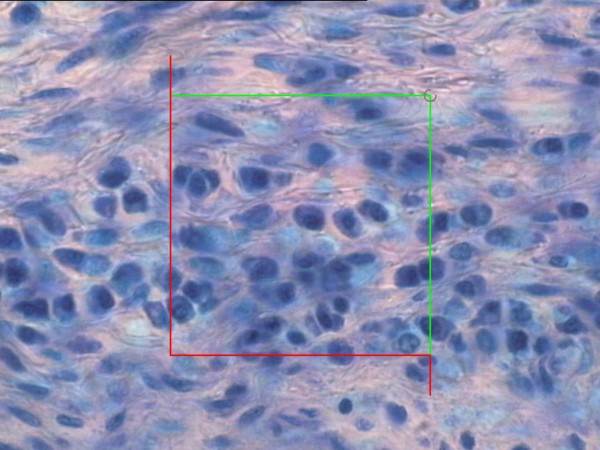
**Typical band of plasma cells seen in the SSCT**. An unbiased counting frame is projected on the section in the CAST 2 computer program; counting is done inside this frame. The criteria for identification of plasma cells were: a round nucleus, typical chromatine staining, and sparse cytoplasmatic staining. (*Toluidin Blue, magnification: ×1540*)

The sections were viewed in an Olympus BX 50 microscope equipped with a Heindenhain VRZ 401 microcater. The microscope was mounted with a microscope stage and a 3 CCD video camera connected to a computer. The stereology software superimposed a 2D-unbiased counting frame on the microscope image using a frame grabber (Flashpoint 3D PRO, Image Processing Solution Inc., North Reading, MA, USA). Sections were studied in a total magnification of 1540. Fields of view were sampled in a systematic and uniformly random manner with X- and Y-steps of 100 μm. The section height was measured uniformly in each section. A disector height of 15 μm was used with an upper and lower exclusion level of 5 μm each. In each field of view an unbiased counting frame with the area of 3440 μm^2 ^was superimposed on the screen (Figure 3).

### Statistics

To compare inter-group differences on continuing data one-way analyses of variance tests were applied. Because of a value of zero in the plasma cell counting, the data were transformed to log(x+2), which also makes the data normally distributed.

In the volume and number estimates, the variance of the method was calculated as the coefficient of error (CE). [21]. The coefficient of variation was calculated for the histomorphometric measurements.

Differences between the groups were tested using Kruskal-Wallis test and the two-sample Wilcoxon rank-sum (Mann-Whitney) test for intergroup differences the semi-quantitative scoring.

The statistics program Stata (Intercooled Stata 8.2, StataCorp, College Station, TX, USA) was used. In all calculations a *p *<*0.05 *was considered significant.

## Results

Thirty-two animals were available for histological investigation after completion of the study. Ten animals were lost due to the antigen-induced anaphylactic reaction. Additionally, one animal from the systemic etanercept group was excluded from the calculations as an outlier because of a very high number of plasma cells in the SSCT of more than 3 standard deviations above the second highest. The high cellular count may be explained by an intra-articular infection. However, neither neutrophils nor bacteria were observed in the SSCT. Only animals completing the entire study were used in the statistical evaluation.

**Figure 4:**
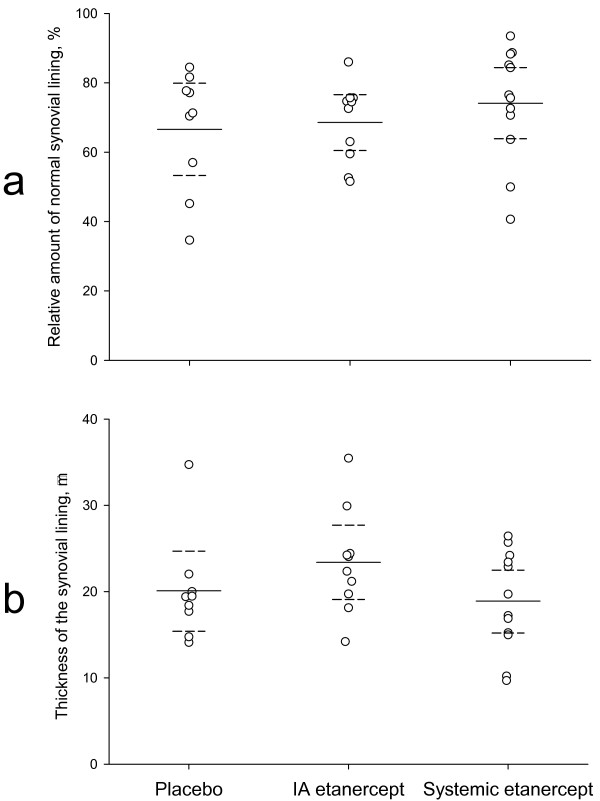
**Histomorphometric results**. The dotted lines are the 95% confidence intervals. **a: **Fractional surface measurements. No statistical differences are seen between the groups. **b: **Synovial lining thickness. No statistical differences are seen between the groups.

Data from the histomorphometry are shown in Figure 4. Neither the fractional surface nor the thickness of the synovial lining changed after treatment with either IA or subcutaneous etanercept.

**Figure 5:**
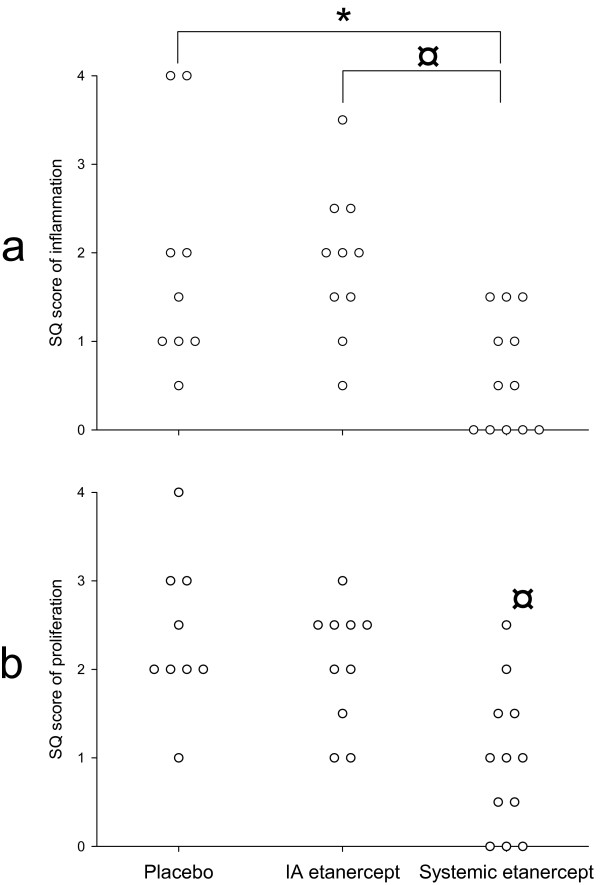
**Semi-quantitative scores**. a: S-Q scores of inflammation. Scoring criteria: 0 = no plasma cells; 1 = few, scattered; 2 = clearly present; 3 = multiple plasma cells organized in bands; 4 = massive presence of plasma cells. b: S-Q scores of synovial proliferation. Scoring criteria: 0 = none; 1 = small areas with synovial proliferation; 2 = large areas with synovial proliferation; 3 = invasion into the joint cavity by synovial proliferation; 4 = joint cavity totally occupied by synovial proliferation. A score was made of both the inner and outer synovial linings and the combined score was divided by two. ¤: p < 0.01, *: p < 0.05.

The semi-quantitative scoring of inflammation in the joint revealed that the systemic etanercept group had significantly lower scores (p = 0.009) using an ANOVA test. When t-tests were done they showed significant differences to the saline (p = 0.013) and the IA etanercept group (p = 0.0017), respectively (Figure 5a). Figure 5b shows the synovial proliferation scores. The systemic etanercept group scores lowest with a p = 0.0025 against the saline group and p = 0.0061 against the IA etanercept group.

**Figure 6:**
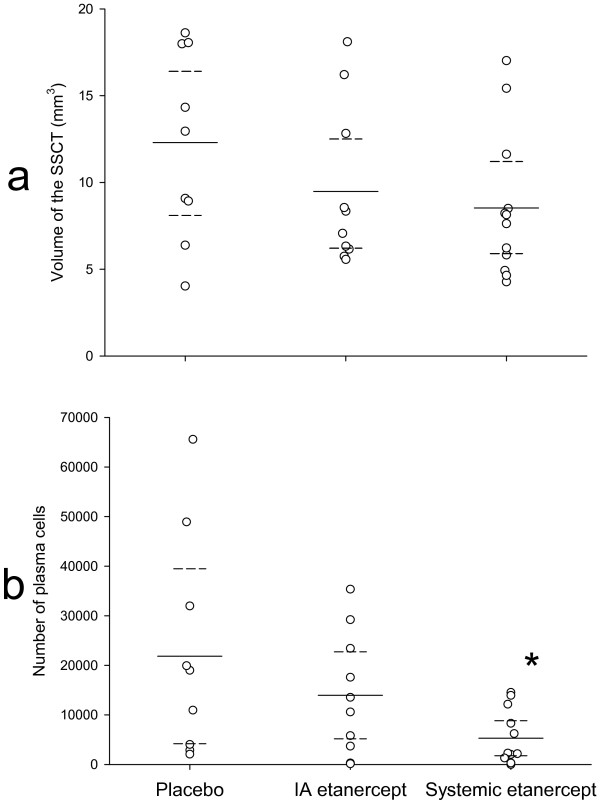
**Stereological estimates**. **a: **The volume of the SSCT. Dotted lines indicate the 95% confidence intervals. No significant differences are seen between the groups. **b: **The number of plasma cells in the SSCT estimated by the optical fractionator. Dotted lines indicate the 95% confidence intervals. *: p < 0.05.

No differences in volume of the SSCT were seen between the groups (Figure 6a). The number of plasma cells in the SSCT was significantly lower in the systemic etanercept group compared to both the placebo and IA etanercept groups with an ANOVA test result of p = 0.03 and t-tests of p = 0.04 between the groups, respectively (Figure 6b).

### Intra-observer variance

Conventional histological measurements: The coefficient of variation was 0.042. Cell counting: The coefficient of variation was 0.12. This is an acceptable value because of the relatively few and scattered cells in the sections [22].

Stereology: The *CE(N) *value was 0.053. The coefficient of error for cell counting was only calculated when the number of cells > 10,000 due to the low number and scattered positions of the cells in the sections [23]. *CE(Pi) *value of the Cavalieri estimator was 0.03.

## Discussion

Inflammation in the TMJs in young rabbits was induced and maintained for 12 weeks by a modified method originally described by Kapila et al. [16]. The model causes inflammatory changes in the TMJ and contains both an acute and a chronic stage predominated by mononuclear cells [24,25], which is characteristic of the chronic inflammation in patients with JIA [26] or rheumatoid arthritis [27]. Thus, the histological features of this model resemble the conditions seen in both rheumatoid arthritis and JIA. The ovalbumin-induced arthritis has been thoroughly tested and used for more than 40 years, but as with other experimental models they rarely each reflect the full extent of the complex, multi-faceted inflammatory process seen in human autoimmune disease [28,29]. One must therefore always be careful when extrapolating data from animal studies to human studies.

TNF-α contributes to joint damage by its diverse actions on proinflammatory cells and cytokines including the receptor activator for nuclear factor-κB ligand (RANKL) pathway [30]. TNF-α is elevated in both serum and synovial fluid of children with juvenile idiopathic arthritis [31]. Also, high levels of TNF-α present in synovial fluid from the TMJ are related to increased TMJ pain experience in rheumatoid arthritis patients [32]. Blocking TNF-α by using subcutaneous etanercept has proven its efficacy and safety for the treatment of polyarticular JIA [12,13]. Therefore, intra-articular blockade of TNF-α could be a possible therapy for patients with arthritis in the TMJ.

Intra-articular corticosteroids improves jaw movement and reduces symptoms in arthritic temporomandibular joints in children with juvenile idiopathic arthritis [8,33]. However, the long-term effects of repeated IA corticosteroids on the mandibular growth are still unknown. In previous studies we have examined IA corticosteroids in antigen-induced TMJ arthritis in rabbits. Although reducing TMJ inflammation, IA corticosteroids aggravates the arthritis-induced inhibition of the mandibular growth [9-11].

The systemically administered etanercept was given once weekly (0.8 mg/kg), which is equivalent to the human dose per week given for the treatment of JIA [34,35]. The etanercept dose given intra-articularly (0.1 mg/kg per joint) was adjusted from a human study [36]. However, no strict regimen for IA etanercept treatment exists [37], and it can be argued that a higher dose should be given. There are currently no studies describing the dose/response of etanercept in rabbits. However, one study reports that even high doses of etanercept is well tolerated in healthy rabbit eyes where a dose of 1 mg/kg was administered [38]. This implies that higher doses also can be given IA without toxic effects.

The model is essentially based on antigenic stimulation by ovalbumin, which causes a systemic activation of the immune system, proliferation of B-cells which are transformed to plasma cells releasing antibodies in the synovial tissues. Therefore, because systemic administration with the highest accumulated dose has the greatest potential of suppressing the number of plasma cells systemically. Herein lays a bias in this model when comparing systemic and local drug administration when evaluating the number plasma cells. But what we can see is that local administration of etanercept does not lower the number of plasma cells compared to saline injections, meaning that the IA etanercept does not lower the migration of plasma cells to the inflamed joint. Using this model on rabbit knees Idogawa and co-workers showed that the TNF-production is high in the joint initially after ovalbumin injection and suggested that the inflammation is TNF-driven. However, our IA etanercept injections were performed seven days after ovalbumin injection where the inflammation is chronic. This may explain why the effects of the IA etanercept are low; we speculate that the effect on initial inflammation can be higher than in chronic antigen-induced arthritis.

The lack of effect of IA etanercept could also be explained by its pharmacokinetics. The distribution of etanercept is rapid, and it will therefore quickly diffuse out of the joint. With a half-life between 70.8 and 96.4 hours in the organism it is possible that the IA concentration is rather low after injection [35]. The time the etanercept stays within the joint is not known. Also, the accumulated total dose given is twelve times higher in the systemic group and in combination with the rapid distribution of etanercept this could be the reason for the lack of effect of IA etanercept. Applying the etanercept intra-articularly too often could result in scar-tissue formation and is not advised.

The results of the fractional surface measurements did not reveal differences between the groups (Figure 4a). A large percentage of the synovial lining is normal in all groups, and a fractional surface measurement does not categorize the villous hyperplasia or the hyperplastic synovium according to degree, but merely as being present or not. However, when given scores, clear statistical evidence of lower scores in the systemic etanercept group compared to the other groups was seen (Figure 5b). Therefore, systemic etanercept has favorable immunomodulatory effects on the synovial lining in this model.

The thickness of the synovial lining was no different between the groups (Figure 4b). This result was also found in our previous study using IA corticosteroid [9].

Semi-quantitative scoring of the inflammation in the SSCT revealed reduced inflammation in the systemic etanercept group. S-Q scores (Figure 5a) and estimation of the number of plasma cells in the SSCT (Figure 6b) both indicate that the chronic inflammation is effectively reduced in the rabbit.

No difference between the saline and IA etanercept groups was present, neither in scoring of the synovial lining nor the SSCT. Previously, we have found that IA saline injections did not cause inflammation nor accumulation of plasma cells in the non-pathological SSCT [9]. Therefore, because the S-Q scores and synovial lining measurements were similar for the IA saline and IA etanercept groups, we conclude that IA etanercept has no significant effect on the parameters studied in this study of chronic inflammation.

## Conclusion

Although numerous cytokines are involved in the pathogenesis of JIA [31], TNF-α-blockade by the use of etanercept alone had significant anti-inflammatory effects on experimentally ovalbumin-induced TMJ arthritis. However, in the parameters used in this study IA etanercept (0.1 mg/kg) had no significant effects on the severity of inflammation in ovalbumin antigen-induced arthritis and was inferior to systemic administration of etanercept in management of chronic inflammation.

## Abbreviations

JIA: Juvenile Idiopathic Arthritis; TMJ: Temporomandibular Joint; IA: Intra-articular; TNF: Tumor Necrosis Factor; S-Q: Semi-quantitative; SSCT: Sub-synovial Connective Tissue; CE: Coefficient of Error; RANKL: Receptor Activator for Nuclear Factor-κB Ligand.

## Competing interests

The authors declare that they have no competing interests.

## Authors' contributions

KDK, PS, AK, TKP and TH designed and conducted the experiment. KDK, EH, AK and JRN decided upon the histological methods to be used. KDK did the histomorphometric, stereological data collection and statistics under supervision by AK and JRN. EH conducted the semi-quantitative assessments. KDK drafted the initial manuscript. All authors have made significant contributions to the manuscript regarding content and interpretation and read and approved the final manuscript.
